# The relationship of ethnicity to the prevalence and management of hypertension and associated chronic kidney disease

**DOI:** 10.1186/1471-2369-12-41

**Published:** 2011-09-06

**Authors:** Sally Hull, Gavin Dreyer, Ellena Badrick, Alistair Chesser, Muhammad Magdi Yaqoob

**Affiliations:** 1 Centre for health sciences, Queen Mary University,London, UK; 2 Renal Department, Barts and the London NHS trust, London, UK

**Keywords:** blood pressure, chronic kidney disease, eGFR, hypertension, ethnicity

## Abstract

**Background:**

The effect of ethnicity on the prevalence and management of hypertension and associated chronic kidney (CKD) disease in the UK is unknown.

**Methods:**

We performed a cross sectional study of 49,203 adults with hypertension to establish the prevalence and management of hypertension and associated CKD by ethnicity. Routinely collected data from general practice hypertension registers in 148 practices in London between 1/1/07 and 31/3/08 were analysed.

**Results:**

The crude prevalence of hypertension was 9.5%, and by ethnicity was 8.2% for White, 11.3% for South Asian and 11.1% for Black groups. The prevalence of CKD stages 3-5 among those with hypertension was 22%. Stage 3 CKD was less prevalent in South Asian groups (OR 0.77, 95% CI 0.67 - 0.88) compared to Whites (reference population) with Black groups having similar rates to Whites. The prevalence of severe CKD (stages 4-5) was higher in the South Asian group (OR 1.53, 95% CI 1.17 - 2.0) compared to Whites, but did not differ between Black and White groups. In the whole hypertension cohort, achievement of target blood pressure (< 140/90 mmHg) was better in South Asian (OR 1.43, 95% CI 1.28 - 1.60) and worse in Black groups (OR 0.79, 95% CI 0.74 - 0.84) compared to White patients. Hypertensive medication was prescribed unequally among ethnic groups for any degree of blood pressure control.

**Conclusions:**

Significant variations exist in the prevalence of hypertension and associated CKD and its management between the major ethnic groups. Among those with CKD less than 50% were treated to a target BP of ≤ 130/80 mmHg. Rates of ACE-I/ARB prescribing for those with CKD were less than optimal, with the lowest rates (58.5%) among Black groups.

## Background

Hypertension is a major risk factor for cardiovascular disease (CVD) and for progression of chronic kidney disease (CKD) towards end stage kidney disease (ESKD) [1,2]. The public health burden of CKD and ESKD is a matter of national priority in the UK [3] and is the subject of recently published national CKD guidelines [4]. The seventh report from the Joint National Committee on Prevention, Detection, Evaluation, and Treatment of High Blood Pressure (JNC 7) and the National Kidney Foundation Kidney Disease Outcomes Quality Initiative (NKF KDOQI) [1,5] have identified patients with CKD as a high risk group in which blood pressure control is particularly important. Studies in the USA have identified that blood pressure control in CKD is poor and that risk factors for poor blood pressure control in patients with CKD include non-Hispanic Black ethnicity, albuminuria and age > 75 [6]. Similarly in the UK it is of concern that a higher proportion of the prevalent renal replacement population comes from ethnic minority groups compared to the UK Caucasian population (17.8% vs 11%) [7].

The introduction of performance related pay for general practice, the quality and outcomes framework (QOF) in 2004 [8] has contributed to the improvement in hypertension control in the UK, and there is also evidence that inequalities in performance between affluent and deprived areas have diminished [9,10]. However there remains room for improvement, especially in high risk groups such as individuals with CKD [11].

In the UK, the effect of ethnicity on blood pressure control and associated CKD in a hypertensive population is not known. Previous studies of primary care hypertension management in the UK have excluded or had very low numbers of patients with CKD [9,11] or have had small ethnic minority populations [11,12]. Millet et al [9,13] have demonstrated disparities by ethnicity in blood pressure control in a London study, but had low reporting rates of CKD.

This study uses data from a large, multi-ethnic population with hypertension and high recorded levels of self reported ethnicity. We have excluded patients with a diagnosis of diabetes mellitus, as this group has previously been the subject of a related study [14]. Our aim is to examine the effect of ethnicity on the prevalence and management of hypertension, and on the prevalence and severity of associated CKD.

## Methods

The study was set in the three geographically contiguous east London Primary Care Trusts (PCTs) of Newham, Tower Hamlets and City & Hackney, with a combined GP registered population of 829,710 in mid 2008. In the 2001 UK census 51.3% of the population in these three PCTs was recorded as of non-White ethnic origin [15] (see Additional File 1: Table S1/S2 for complete demographic breakdown). The populations of these PCTs are among the eight most socially deprived localities in Britain [15]. 148 of the 151 general practices contributed data as part of a regular annual audit of chronic disease management, which forms part of a primary care chronic disease management programme [16]. Practice data covered more than 98% of the GP registered population in the three PCTs. All data was fully anonymised, and managed according to UK NHS information governance requirements. Ethics approval was not required for this observational study.

### Clinical data collection

Practice computer databases were interrogated using Morbidity Information Query and Export Syntax (MIQUEST) software which has been validated as an accurate and appropriate method for data extraction of patients with CKD in general practice [17]. All adult patients with a computerised diagnostic Read code for hypertension were included. Read codes are the clinical classification system used in UK general practice to identify patients with a specific chronic disease [18]. Variables included in the analysis were recorded in the period 1/1/07 and 31/3/08. While patients may have had multiple clinic visits during this period, only the latest data point within the study period was used for analysis. Variables for analysis included renal function as the estimated glomerular filtration rate (eGFR) expressed by the 4 variable MDRD equation [19], systolic and diastolic blood pressure, total cholesterol, smoking status, diagnosis of ischaemic heart disease, and prescription data for the use of antihypertensive medications (angiotensin converting enzyme inhibitor (ACE-I), angiotensin receptor blocker (ARB), thiazide diuretics, calcium channel blockers, beta blockers and alpha blockers). Blood pressure was measured using both manual and automated devices during routine clinical consultations. Data entry to the hypertension register occurs during the consultation.

Isolated systolic hypertension (ISH) is defined as stage 1 ≥ 140 and ≤ 90 mmHg and stage 2 as ≥ 160/≤ 90 mmHg. Renal function is expressed as stage of chronic kidney disease defined by current UK guidelines [19] based on the eGFR value: stage 3a CKD (eGFR 45-59 ml/min/1.73 m^2^), stage 3b CKD (eGFR 30-44 ml/min/1.73 m^2^), stage 4 CKD (eGFR 15-29 ml/min/1.73 m^2^) and stage 5 CKD (eGFR < 15 ml/min/1.73 m^2^). All eGFR measures have been corrected for Black ethnicity (multiply eGFR by 1.21). Different methods for measuring the serum creatinine (from which the eGFR is calculated) are used in the study area, however, as we report elsewhere [14], differences between techniques result in very small changes in eGFR which are likely to be equally distributed across the different ethnic groups and we do not consider that these differences will change the overall classification of CKD.

During the study period, of the 75,103 adults coded with hypertension, 92.4% had ethnicity recorded. We excluded the 28% with a diagnosis of diabetes (see Additional File 1: Table S3/S4 for analysis with diabetic cases included), leaving 49,203 cases with hypertension for analysis. An eGFR (recorded in the fifteen month study period) was available for analysis in 57.1% of cases. The proportion of cases with an eGFR measure varied by ethnic group (White 62%, South Asian 54.6%, Black 52.3%, Chi^2 ^p < 0.001). A blood pressure reading was recorded for 92%, total cholesterol for 73%, and smoking status for 80.7%. Due to low levels of recorded proteinuria (5%) and body mass index (BMI) (15%) during the study we excluded these variables from the analysis.

### Ethnicity data

Self reported ethnicity was recorded at the practice during registration or routine consultation [14]. Ethnic categories are based on the UK 2001 census and for this study were condensed into five categories: White (British, Irish, other White), Black (Black African, Black Caribbean, Black British, other Black and mixed Black), South Asian (Bangladeshi, Pakistani, Indian, Sri Lankan, British Asian, other South Asian or mixed Asian), Other (Chinese, Other ethnic groups, other mixed groups), and unknown. Missing or incomplete data was recorded as unknown (see Additional File 1: Table S1 for full details of UK 2001 Census ethnic groups).

To calculate the prevalence of hypertension in each ethnic group in the study area, we used the Greater London Authority (GLA) mid-year 2006 age banded population estimates by ethnic group [20]. The GLA provides estimates of ethnic populations by age band, which are adjusted from the Office of National Statistics (ONS) midyear estimates. Although the ONS data are considered accurate for London overall, they conceal under counting for some inner London boroughs such as Newham. The GLA produce a 'high' and 'low' population estimates, both estimates produced similar hypertensive prevalence figures, our report uses the low estimate as population denominator.

### Statistical analysis

All statistical analyses used Stata version 10 [21]. Differences in the prevalence of CKD between ethnic groups are expressed as odds ratios with White ethnicity considered as neutral risk (odds ratio = 1). Age and sex adjusted odds ratios for CKD prevalence were adjusted by key risk factors (mean systolic blood pressure, mean total cholesterol, smoking status and IHD diagnosis) and clustered by GP practice. Blood pressure control is expressed as a percentage of those meeting previously defined values for either poor or optimal control in each group. Categorical variables were assessed for differences by using a Chi squared test, continuous numerical variables using Anova.

## Results

**Figure 1:**
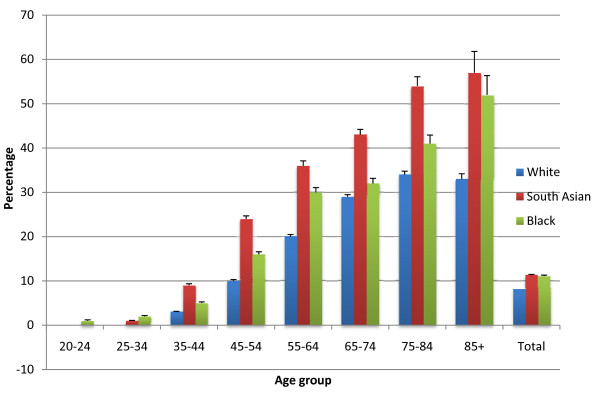
**Prevalence of essential Hypertension by age and aggregated ethnic groups, using low east London GLA population estimates as denominator**. Values are population percentages (95% CIs).

In this cohort 45.3% were males, the average age for males was 59.7, and for females was 62.4 years. The crude prevalence of hypertension among adults in east London was 9.5% and by ethnic group was 8.2% for White, 11.3% for South Asian and 11.1% for Black groups. East London has a very young population, the crude population prevalence rises to 14.8% when age standardized to the European standard population. The prevalence of hypertension by age and ethnicity was examined (removing those with diagnosed diabetes mellitus), using practice data as numerator, and the midyear population estimates from the Greater London Authority as denominator. Figure 1 illustrates the trend towards earlier onset and higher prevalence of hypertension among ethnic minority groups, with the South Asian groups having the highest prevalence of hypertension among all age groups.

The crude prevalence of CKD (eGFR < 60 ml/min/1.73 m^2^) among the hypertensive population was 22%. The prevalence of CKD risk factors by ethnicity and gender was examined. The White group had a higher mean age and smoking rates than other groups (p < 0.001). Black groups had the lowest rates of recorded ischaemic heart disease (p < 0.001). The distribution of risk factors for CKD by ethnicity, and the prevalence of CKD among all those with an eGFR value are shown in tables 1 and 2.

**Table 1:** Prevalence of CKD risk factors among all hypertensives by ethnicity and gender (excluding those with diabetes mellitus)

	No. of individuals	Mean age (years)	SBP Mean (SD) mmHg	DBP Mean (SD) mmHg	Total cholesterol Mean (SD) mmol/L	% with IHD diagnosis	% smoking
**White**	22 489	65.2	136.5 (16.5)	79.1 (10.6)	4.9 (1.1)	13.8	24.2

**South Asian**	9 757	57.4	132.6 (16.7)	79.7 (10.4)	4.7 (1.1)	11.6	14.2

**Black**	12 717	57.0	138.3 (16.9)	83.0 (11.0)	4.9 (1.0)	5.1	12.7

**Other**	3 776	62.0	136.3 (16.5)	80.3 (10.8)	5.0 (1.1)	12.6	17.5

	p † =	< 0.001	< 0.001	< 0.001	< 0.001	< 0.001	< 0.001

**Female**	27 018	62.4	135.9 (16.7)	79.9 (10.5)	5.0 (1.1)	8.0	17.1

**Male**	22 185	59.9	137 (17.0)	81.1 (11.2)	4.7 (1.1)	13.8	21.6

* Abbreviations: SBP - systolic blood pressure, DBP diastolic blood pressure, IHD - ischaemic heart disease

† - p value for difference between groups (ANOVA)

**Table 2:** Prevalence of CKD stages 3-5 among hypertension cases with eGFR values (ml/min/1.73 m^2^), by ethnicity and gender (excluding those with diabetes mellitus)

	Hypertensives with eGFR measured (n)*†	eGFR > 60	CKD 3a eGFR 45-60	CKD 3b eGFR 30-45	CKD 4 eGFR 15-30	CKD 5 eGFR < 15
**White**	13 953	75.6%	16.7%	6.2%	1.3%	0.2%

**South Asian**	5 324	85.6%	9.8%	2.9%	1.1%	0.6%

**Black**	6 731	81.8%	14.3%	2.8%	0.7%	0.5%

**Other**	1 859	80.7%	13.0%	4.9%	1.2%	0.2%

						

**Female**	16 267	76.5%	16.5%	5.3%	1.1%	0.3%

**Male**	13 482	82.5%	12.0%	3.9%	1.1%	0.5%

* eGFR (ml/min/1.73 m^2^) recorded in the preceding 15 months

†The proportion of cases with a valid eGFR varied by ethnic group (White 62%, South Asian 54.6%, Black 52.3%, p < 0.001).

Using logistic regression we first examined the prevalence of stage 3 CKD (eGFR 30-59 ml/min/1.73 m^2^) by ethnicity among all hypertensives, adjusted for age and sex and clustered by practice. Compared to the White population the South Asian population had a significantly lower overall prevalence of CKD stage 3 (OR 0.77, 95% CI: 0.67-0.88) and this difference remains with further adjustment for systolic blood pressure, total cholesterol, smoking status and diagnosis of ischaemic heart disease (see Table 3).

**Table 3:** Ethnicity and the prevalence of CKD stage 3 (eGFR 30-59 ml/min/1.73 m^2^) among hypertensive patients

Ethnic group	No. hypertensive	No. hypertensive with CKD 3 only	CKD stage 3* Odds Ratio (95% CI)	**CKD stage 3 **† **Odds Ratio (95% CI)**
**White**	13 953	3 193	1	1

**South Asian**	5 324	681	0.77 (0.67-0.88)	0.75 (0.64-0.88)

**Black**	6 731	1 147	1.13 (1.01-1.28)	1.09 (0.95-1.25)

**Other**	1 859	333	0.85 (0.74-0.98)	0.78 (0.68-0.90)

*adjusted by age and sex and clustered by GP practice

† *additional adjustment by systolic blood pressure, total cholesterol, smoking status and diagnosis of IHD*

We then examined the risks of having severe CKD (stages 4 and 5, eGFR < 30 ml/min/1.73 m^2^), by conducting a further analysis restricted to cases with an eGFR < 60 ml/min/1.73 m^2 ^to determine if ethnicity is marker for disease severity in established cases of CKD. This showed that South Asian groups had a significantly greater risk of severe CKD (stages 4-5, eGFR < 30 ml/min/1.73 m^2^) compared to the White population (OR 1.53, 95% CI: 1.17-2.0 with adjustment for risk factors) (see Table 4).

**Table 4:** Ethnicity and the prevalence of CKD stage 4 and 5 among hypertensives with an eGFR < 60 ml/min/1.73 m^2^

Ethnic group	No. hypertensive	No. hypertensive with CKD 4 and 5	CKD stages 4,5* OR (95% CI)	**CKD stages 4,5 **† OR (95% CI)
**White**	3 407	214	1	1

**South Asian**	767	86	1.53 (1.17, 2.0)	1.44 (1.00, 1.85)

**Black**	1 223	76	0.80 (0.60, 1.06)	0.87 (0.62, 1.22)

**Other**	359	26	1.10 (0.77, 1.58)	1.12 (0.75, 1.47)

*adjusted by age and sex and clustered by GP practice

† *additional adjustment by systolic blood pressure, total cholesterol, smoking status and diagnosis of IHD*

Table 5 shows blood pressure control by ethnic group, comparing control among hypertensive patients with eGFR values above and below 60 ml/min/1.73 m^2^. 14.4% of patients had blood pressure above the target value of 140/90 mmHg. Those with Black ethnicity had worse blood pressure control compared to other groups, regardless of CKD status. South Asian groups achieved the best blood pressure control. This was confirmed by further analysis to examine the effect of ethnicity on the achievement of controlled blood pressure (< 140 and < 90 mmHg) regardless of CKD status, adjusted by age and sex and clustered by practice. Achievement of target blood pressure control was better in South Asian (OR 1.43, 95% CI 1.28 to 1.60) and worse in Black groups (OR 0.79, 95% CI 0.74 to 0.84) compared to the White population.

**Table 5:** Blood pressure control among hypertensives with and without CKD, by ethnicity

Ethnic group	No. of hypertensives	% with BP > 140 and > 90 mmHg	% with BP ≤130 and ≤80 mmHg	Mean no of anti-hypertensive drugs
**All hypertensives**				

**White**	21 113	11.9	28.9	1.78

**South Asian**	8 839	12.2	37.5	1.62

**Black**	11 791	20.4	24.2	1.95

**Chi squared test, p = **		< 0.001	< 0.001	< 0.001

**eGFR > 60 ml/min/1.73 m^2^**				

**White**	10 353	11.7	27.2	1.81

**South Asian**	4 469	11.9	35.9	1.64

**Black**	5 418	19.5	23.8	2.04

**Chi squared test, p = **		< 0.001	< 0.001	< 0.001

**eGFR < 60 ml/min/1.73 m^2^**				

**White**	3 365	6.63	34.1	2.16

**South Asian**	756	8.07	42.7	2.00

**Black**	1 202	14.3	28.2	2.38

**Chi squared test, p = **		< 0.001	< 0.001	< 0.001

The mean number of prescribed medications for hypertension for those without CKD was 1.83, and for those with CKD was 2.17. Among those with CKD less than 50% were treated to a target BP of ≤ 130/80 mmHg. A higher proportion of South Asian patients (42.7%) achieved this target than other ethnic groups. Among this hypertensive population only 3.2% had stage 2 ISH (SBP > 160 and DBP < 90 mmHg).

Table 6 shows prescribing rates stratified by blood pressure control. There are marked differences in prescribing by ethnicity, with lower rates of ACE-I/ARB prescribing for Black groups, but higher rates of calcium channel blocker and thiazide diuretic use in this population, commensurate with current guidelines [22]. Black groups are prescribed more medications at all levels of blood pressure control.

**Table 6:** Prescribing rates by ethnicity, stratified by BP control

	No. of hypertensives	Mean Blood pressure mmHg (SD)	% ACE/ARB	% CCB	% Thiazides	% Beta-blockers	% Alpha blockers	Mean no. of drugs
**BP > 140 and** **> 90 mm Hg**								

**White**	6 567	152.4 (13.1)	54.6	54.2	45.7	28.6	12.6	1.95

**South Asian**	8 839	152.4 (13.9)	52.2	54.6	37.5	27.5	9.5	1.81

**Black**	11 791	153.6 (14)	45.7	74.5	51.1	23.3	17.9	2.13

**ANOVA p value**			< 0.01	< 0.01	< 0.01	< 0.01	< 0.01	< 0.01

**BP < 130 and < 80 mmHg**								

**White**	6 110	128.2 (14.2)	52.7	46.2	38.1	31.6	9.4	1.78

**South Asian**	3 318	125.8 (14)	48.6	47.0	30.0	27.7	7.3	1.60

**Black**	2 855	129.8 (14)	39.2	64.5	49.8	25.6	11.6	1.90

**ANOVA p value**			< 0.01	< 0.01	< 0.01	< 0.01	< 0.01	< 0.01

* ACE-I - angiotensin converting enzyme inhibitor, ARB - angiotensin receptor blocker, CCB - calcium channel blocker

Table 7 shows prescribing stratified by eGFR level. This shows that patients with CKD were prescribed a higher mean number of antihypertensive drugs. Approximately 60% of hypertensive patients with CKD were prescribed ACE-I/ARB medications.

**Table 7:** Prescribing rates by ethnicity, stratified by eGFR level

	No. of hypertensives	Mean blood pressure mmHg (SD)	% ACE/ARB	% CCB	% Thiazides	% Beta-blockers	% Alpha blockers	**Mean no**. of drugs
**eGFR > 60 ml/min/1.73 m^2^**								

**White**	10 546	136.5 (15.5)	54.3	48.1	41.9	27.1	9.8	1.81

**South Asian**	4 557	132.4 (16.1)	51.5	47.1	32.5	25.5	7.6	1.64

**Black**	5 508	138.2 (16.1)	43.2	71.4	53.0	22.0	14.7	2.04

**ANOVA p value**		< 0.01	< 0.01	< 0.01	< 0.01	< 0.01	< 0.01	< 0.01

**eGFR < 60 ml/min/1.73 m^2^**								

**White**	3 407	136.1 (17.4)	64.2	56.3	35.7	16.2	43.5	2.16

**South Asian**	767	134.3 (18.8)	60.3	56.7	32.7	12.4	37.4	2.00

**Black**	1 223	138.9 (18.4)	58.5	72.9	30.3	22.7	53.7	2.38

**ANOVA p value**		< 0.01	< 0.01	< 0.01	< 0.01	< 0.01	< 0.01	< 0.01

* Abbreviations: ACE-I - angiotensin converting enzyme inhibitor, ARB - angiotensin receptor blocker, CCB - calcium channel blocker

## Discussion

This study is the first of its kind to examine the association of ethnicity, hypertension and CKD in a UK population. Using routine clinical data from a socially deprived multiethnic population with uniquely high levels of self reported ethnicity, we examine variation in prevalence and management of hypertension and associated CKD. The exclusion of patients with diabetes mellitus, which has much higher rates among the South Asian population, allows us to evaluate the effect of hypertension on CKD in isolation (see Additional File 1: Table S3/S4 for an analysis including diabetic patients). Our results illustrate the earlier onset and higher prevalence of hypertension among South Asian and Black groups compared to the White population in the UK. In our cohort the prevalence of CKD stages 3-5 was 22%. This is higher than the 18% prevalence of CKD identified in a diabetic cohort in the same area of the UK, suggesting that hypertension may be an equally powerful risk factor for the development of CKD as diabetes mellitus [14]. White groups were associated with a higher prevalence of milder CKD (stage 3) (see table 3). In common with our findings for diabetic patients, severe CKD (stages 4,5) is significantly more prevalent among South Asian groups, even after adjustment for age and key clinical indicators.

Blood pressure control in the UK has shown steady improvement over time [11]. Pay for performance schemes, such as the QOF, contribute to this, with evidence suggesting that quality of care for conditions targeted in the UK QOF improve, albeit with a ceiling effect [23]. In this cohort over 80% had BP values =< 140/90 mmHg, which compares favourably with 2006 data from the Health survey for England which reported 52% control to < 140/90 mmHg among those with treated hypertension. We found significant differences by ethnicity in achieving this target, with lower rates among Black groups. High systolic blood pressure is a key determinant of CKD progression [24] and the benefits of treatment for systolic blood pressure have been clearly established [25-27]. Access to primary and secondary care is free at the point of need in the UK NHS and free prescriptions are available for the poorest groups, hence differential access to care in this population is unlikely to account for the observed differences between groups.

In patients with an eGFR < 60 ml/min/1.73 m^2^, a recommended blood pressure of < 130/80 mmHg [1] was achieved in less than 50% of patients in all ethnic groups and less than 30% in Black groups. These findings are comparable with a previous study of diabetic patients with CKD in east London, and a study of patients with CKD in the USA [6,14]. The renoprotective benefits of ACE-I and ARB's are well established [28-30] and current UK CKD guidelines advocate the use of these drugs in patients with CKD [4]. However nearly 40% of patients with CKD in this study did not receive an ACE-I or ARB, and Black patients were significantly less likely to be prescribed these medications than other ethnic groups in contrast to the findings of the recent AASK study which demonstrates the benefits of these medications in this patient group [28].

We found that achievement of target blood pressure to < 140/90 mmHg was superior in patients with an eGFR < 60 ml/min/1.73 m^2 ^compared to those with a eGFR > 60 ml/min/1.73 m^2 ^. Previous studies have demonstrated improved blood pressure control in the presence of two or more comorbidities compared to hypertension alone [9,13]. This suggests that clinicians may adopt a more aggressive approach to blood pressure control if patients are identified as "higher risk" due to other cardiovascular comorbidities. Furthermore, the presence of these comorbidities may influence prescribing decisions which contribute to blood pressure control.

Our finding that South Asian patients have higher rates of more severe CKD, in spite of better blood pressure control than other groups requires further consideration, particularly given that the lower mean blood pressure in this group would be expected to result in a lower prevalence of severe renal disease. There are several potential explanations for this finding, one being that hypertension affects South Asians at a younger age than other ethnicities, and the longer duration of disease may lead to more severe complications such as advanced CKD.

Recently the MYH 9 gene has been identified as a marker for more rapid progression to ESKD in Black patients [31,32] due to non-diabetic kidney disease and it is possible that a similar genetic predisposition to more rapid progression even in the face of a relatively low blood pressure exists in the South Asian population but this hypothesis requires further study. An additional explanation for the observed higher prevalence of severe CKD in South Asians may in part be due to more rapid progression of CKD in the South Asian population, combined with increased length of survival in this group. A prospective study examining progression of CKD between different ethnic groups is currently underway to address this issue.

Geographic variations in cardiovascular mortality at a similar blood pressure level are well documented [33]. The uniform blood pressure targets recommended by hypertension guidelines may paradoxically disadvantage some ethnic groups in which the normal mean blood pressure is lower than in other groups. For example if the normal mean blood pressure of South Asians is lower than for Black or White groups, a "one size fits all" blood pressure target may be too high to allow optimal renal protection in South Asian patients.

The strengths of this study include the size of the study population which includes over 50% of patients from ethnic minorities. Our population is likely to be representative of other multiethnic, low income, urban areas in the UK, hence our findings should be generalisable, and of interest to clinicians and health service commissioners. The exclusion of patients with diabetes highlights the higher than expected prevalence of CKD in this patient group with hypertension alone.

The limitations of this study are linked to the pragmatic nature of this primary care database. The entry criteria for a diagnosis of hypertension depends on decisions made in a routine clinical setting, and we use only the latest BP data point for each case for analysis. Similarly the accuracy of comorbid diagnoses, and the completeness of clinical measurements will be subject to routine clinical variation. eGFR values were available for 57% of cases and there was significant variation by ethnic group. We recognise that this may introduce bias. Within the UK health system there are no structural barriers to healthcare, hence differential access to care is unlikely. We are aware that combining sub groups within the South Asian and Black populations may mask important differences in behavior and health beliefs between population groups which in turn may contribute to health outcomes.

We did not have access to individual drug dosing regimes, and so cannot comment on the intensity of hypertension treatment [34] or on whether possible therapeutic inertia by clinicians varied by ethnicity or CKD status. The collection and quantification of proteinuria data in primary care remains suboptimal for hypertension, hence this important variable could not be included in the analysis, eGFR recording is also lower than optimal in this cohort. Neither eGFR nor proteinuria were included as UK QOF indicators for hypertension at the time of data collection which may explain low levels of recording. A cross sectional study such as this cannot take account of deaths and other movement in and out of the cohort, which may affect our overall estimates of the burden of CKD.

Questions also remain about the validation and use of adjustments of the MDRD generated eGFR by ethnicity. Despite these concerns, it is currently in universal use to classify CKD and monitor progression of renal disease. Until more refined equations enter the mainstream, it remains the best tool available to us.

The data from this large cross sectional study identifies differences by ethnicity in the prevalence of hypertension and associated CKD, and in the achievement of blood pressure control and drug prescribing. These disparities in hypertension management have persisted in spite of steady improvements in primary care hypertension management in the UK.

Potential strategies to reduce the observed disparities include improved awareness by clinicians, and the public, of the excess risk associated with certain ethnic groups, and a greater use of ACE-I/ARB and diuretics to improve blood pressure control and reduce progression of kidney disease. The inclusion of annual measures of eGFR, BMI and proteinuria, potentially modifiable risk factors for cardiovascular and progressive renal disease [29,35,36], in the hypertension domain of the UK QOF offers an opportunity to monitor renal function and intervene at an early stage.

## Conclusions

Additional studies need to identify whether target blood pressure for the prevention of CKD progression should to be tailored by reference to ethnicity. Whereas this strategy may have merit at a biological level, any benefit must be measured against the risk of increasing the complexity of blood pressure guidelines. Improving current practice to reach national standards should be the focus of attention while prospective studies are conducted to address some of the unanswered questions.

## Abbreviations

ACE-I: angiotensin converting enzyme inhibito; ARB: angiotensin receptor blocker; BMI: body mass index; BP: blood pressure; CCB: calcium channel blocker; CKD: chronic kidney disease; CVD: cardiovascular disease; eGFR: estimated glomerular filtration rate; ESKD: end stage kidney disease; GLA: greater London authority; ISH: isolated systolic hypertension; JNC: Joint National Committee; MIQUEST: Morbidity Information Query and Export Syntax; NKF KDOQI: National Kidney Foundation Kidney Disease Outcomes Quality Initiative; ONS: office of national statistics; OR: odds ratio; PCT: Primary care trust; QOF: quality and outcomes framework.

## Competing interests

All authors declare that there is no conflict of interest, financial or otherwise, with respect to this study and the above manuscript.

## Authors' contributions

MMY, SH and AC and GD designed the study, EB undertook the analysis. GD and SH led on writing the manuscript with all authors contributing to and reviewing the submitted version. All authors are guarantors for the study. GD and SH contributed equally. All authors read and reviewed the final manuscript

## Pre-publication history

The pre-publication history for this paper can be accessed here:

http://www.biomedcentral.com/1471-2369/12/41/prepub

## Supplementary Material

Additional file 1 **Supplementary demographic information for manuscript - The relationship of ethnicity to the prevalence and management of hypertension and associated chronic kidney disease**. Table 1 contains the demographic comparison between East London and the UK using 2001 census data. Table 2 contains data on the prevalence of hypertension by age and aggregated ethnic groups using low east London GLA population estimates for 2006 as denominator. Tables 3 and 4 show the effect of ethnicity on CKD and hypertension including patients with diabetes mellitus. Click here for file
