# Supplementary material for: The relationship of ethnicity to the prevalence and management of hypertension and associated chronic kidney disease
Source: BMC Nephrol. 2011 Sep 6;12:41. doi: 10.1186/1471-2369-12-41 (PMC3180366; doi:10.1186/1471-2369-12-41)
Supplement: Additional file 1 — Supplementary demographic information for manuscript - The relationship of ethnicity to the prevalence and management of hypertension and associated chronic kidney disease. Table 1 contains the demographic comparison between East London and the UK using 2001 census data. Table 2 contains data on the prevalence of hypertension by age and aggregated ethnic groups using low east London GLA population estimates for 2006 as denominator. Tables 3 and 4 show the effect of ethnicity on CKD and hypertension including patients with diabetes mellitus. [file 1471-2369-12-41-S1.DOC]

**Supplementary information**

**Table S1. Demographic comparison (age and ethnicity) between east London and England based on the 16 categories of the 2001 census**

|  | **East London**  **Hypertensive Cohort**  **Numbers (%)** | **2001 Census data for City and Hackney, Newham, Tower Hamlets, (%)** | **2001 Census data for England (%)** |
| --- | --- | --- | --- |
| **Ethnic Group** |  |  |  |
| All People | 75 183 (100) | 642 821 (100) | 49 138 831 (100) |
|  |  |  |  |
| White British | 23 455 (31.2) | 256 031 (39.8) | 42 747 136 (87.0) |
| White Irish | 1 327 (1.8) | 13 171 (2.0) | 624 115 (1.3) |
| White Other | 4 318 (5.7) | 48 195 (7.5) | 1 308 110 (2.6) |
| Black African | 5 117 (6.8) | 62 868 (9.8) | 475 938 (1.0) |
| Black Caribbean | 2 467 (3.3) | 44 035 (6.9) | 561 246 (1.1) |
| Black Other | 7 176 (9.5) | 8 501 (1.3) | 95 324 (0.2) |
| Asian Bangladeshi | 6 907 (9.3) | 92 981 (14.5) | 275 394 (0.6) |
| Asian Indian | 6 512 (8.7) | 40 222 (6.3) | 1 028 546 (2.1) |
| Asian Pakistani | 2 401 (3.2) | 24 295 (3.8) | 706 539 (1.4) |
| Asian Other | 1 110 (1.5) | 11 025 (1.7) | 237 810 (0.5) |
| Mixed White and Black African | 102 (0.1) | 4 045 (0.6) | 76 498 (0.2) |
| Mixed White and Black Caribbean | 1 940 (2.6) | 7 629 (1.2) | 231 424 (0.5) |
| Mixed White and Asian | 737 (1.0) | 4 576 (0.7) | 184 014 (0.4) |
| Mixed other | 158 (0.21) | 5 372 (0.8) | 151 437 (0.3) |
| Other Chinese | 489 (0.7) | 8 299 (1.3) | 220 681 (0.4) |
| Other | 4 551 (6.1) | 11 576 (1.8) | 214 619 (0.4) |
| Not stated | 619 (0.8) |  |  |
|  |  |  |  |
| Missing data | 5717 (7.6) |  |  |
| **Age Bands** |  |  |  |
| All People | 75 183 | 642 821 (100) | 49 138 831 (100) |
| 0-18 | 40 (0.1) | 174 889 (27.2) | 11 132 847 (22.7) |
| 18-24 | 162 (0.2) | 74 776 (11.6) | 4 130 290 (8.4) |
| 25-34 | 1 610 (2.1) | 134 340 (20.9) | 7 054 271 (14.4) |
| 35-44 | 7 227 (9.6) | 97 248 (15.1) | 7 341 900 (14.9) |
| 45-54 | 15 481 (20.6) | 60 297 (9.4) | 6 494 262 (13.3) |
| 55-64 | 17 427 (23.2) | 42 160 (6.6) | 5 177 261 (10.5) |
| 65-74 | 17 706 (23.6) | 33 015 (5.1) | 4 102 841 (8.3) |
| 75-84 | 11 926 (15.9) | 19 871 (3.1) | 2 751 135 (5.6) |
| 85+ | 3 604 (4.8) | 6 225 (1.0) | 954 024 (1.9) |
|  |  |  |  |

**Table S2. Prevalence of hypertension by age and aggregated ethnic groups using low east London GLA population estimates for 2006 as denominator**

| |  | **White** | **Black** | **South Asian** | **Other** | **TOTAL** | | --- | --- | --- | --- | --- | --- | | 18-24 | 27 084 | 7 888 | 13 634 | 4 346 | 52 953 | |  | 0% | 1% | 0% | 0% | 0% | | 25-34 | 92 413 | 22 689 | 43 347 | 15 249 | 173 698 | |  | 0% | 2% | 1% | 1% | 1% | | 35-44 | 55 951 | 25 902 | 24 605 | 9 744 | 11 6201 | |  | 3% | 5% | 9% | 3% | 5% | | 45-54 | 34 852 | 15 296 | 14 710 | 6 007 | 70 866 | |  | 10% | 16% | 24% | 13% | 15% | | 55-64 | 26 855 | 7 002 | 7 189 | 3 328 | 44 373 | |  | 20% | 30% | 36% | 30% | 25% | | 65-74 | 17 342 | 6 169 | 6 405 | 1 473 | 31 390 | |  | 29% | 32% | 43% | 55% | 34% | | 75-84 | 14 178 | 2 499 | 2 161 | 582 | 19 420 | |  | 34% | 41% | 54% | 99% | 39% | | >=85 | 5 864 | 497 | 409 | 149 | 6 919 | |  | 33% | 52% | 57% | 149% | 38% | | TOTAL | 274 539 | 87 942 | 112 460 | 40 878 | 51 5820 | |  | 8.20% | 11.10% | 11.30% | 9.20% | 9.50% | |  |  |  |  |  |  |  |  |  |  |  |  |  |  |  |  |  |  |
| --- | --- | --- | --- | --- | --- | --- | --- | --- | --- | --- | --- | --- | --- | --- | --- | --- | --- | --- | --- | --- | --- | --- | --- | --- | --- | --- | --- | --- | --- | --- | --- | --- | --- | --- | --- | --- | --- | --- | --- | --- | --- | --- | --- | --- | --- | --- | --- | --- | --- | --- | --- | --- | --- | --- | --- | --- | --- | --- | --- | --- | --- | --- | --- | --- | --- | --- | --- | --- | --- | --- | --- | --- | --- | --- | --- | --- | --- | --- | --- | --- | --- | --- | --- | --- | --- | --- | --- | --- | --- | --- | --- | --- | --- | --- | --- | --- | --- | --- | --- | --- | --- | --- | --- | --- | --- | --- | --- | --- | --- | --- | --- | --- | --- | --- | --- | --- | --- | --- | --- | --- | --- | --- | --- | --- | --- | --- | --- | --- | --- | --- | --- | --- |
|  |  |  |  |  |  |  |  |  |  |  |  |  |  |  |  |  |  |  |
|  |  |  |  |  |  |  |  |  |  |  |  |  |  |  |  |  |  |  |
|  |  |  |  |  |  |  |  |  |  |  |  |  |  |  |  |  |  |  |
|  |  |  |  |  |  |  |  |  |  |  |  |  |  |  |  |  |  |  |
|  |  |  |  |  |  |  |  |  |  |  |  |  |  |  |  |  |  |  |

**Table S3. The effect of ethnicity on the prevalence of CKD stage 3 (eGFR 30-59 ml/min/1.73m^2^) among hypertensive patients adjusted by age and sex and risk factors for severity and progression**

This analysis includes data from the 25 900 patients in the sample coded with diabetes mellitus

| **Ethnic group** | **No. of hypertensives** | **CKD stage 3* Odds Ratio (95% CI)** | **CKD stage 3** † **Odds Ratio (95% CI)** |
| --- | --- | --- | --- |
| *White* | 18 553 | 1 | 1 |
| *South Asian* | 9 825 | 0.82 (0.73-0.93) | 0.81 (0.71-0.91) |
| *Black* | 10 046 | 1.13 (1.03-1.24) | 1.11 (1.00-1.23) |
| *Other* | 2 678 | 0.86 (0.77-0.97) | 0.83 (0.74-0.92) |

**adjusted by age and sex and clustered by GP practice*

† *additional adjustment by systolic blood pressure total cholesterol smoking and diagnosis of IHD*

**Table S4. The effect of ethnicity on the severity of CKD among hypertensives with an eGFR < 60ml/min/1.73m^2^.**

This analysis includes data from the 25 900 patients in the sample coded with diabetes mellitus.

| **Ethnic group** | **No. hypertensives** | **CKD stages 4 5***  **OR (95% CI)** | **CKD stages 4 5**†  **OR (95% CI***)* |
| --- | --- | --- | --- |
| *White* | 4 634 | 1 | 1 |
| *South Asian* | 1 649 | 1.71 (1.39 2.11) | 1.53 (1.21-1.895) |
| *Black* | 2 059 | 0.94 (0.77 1.14) | 1.02 (0.782-1.27) |
| *Other* | 543 | 1.19 (0.93 1.53) | 1.10 (0.84-1.43) |

**adjusted by age and sex and clustered by GP practice*

† *additional adjustment by systolic blood pressure total cholesterol smoking and diagnosis of IHD*
